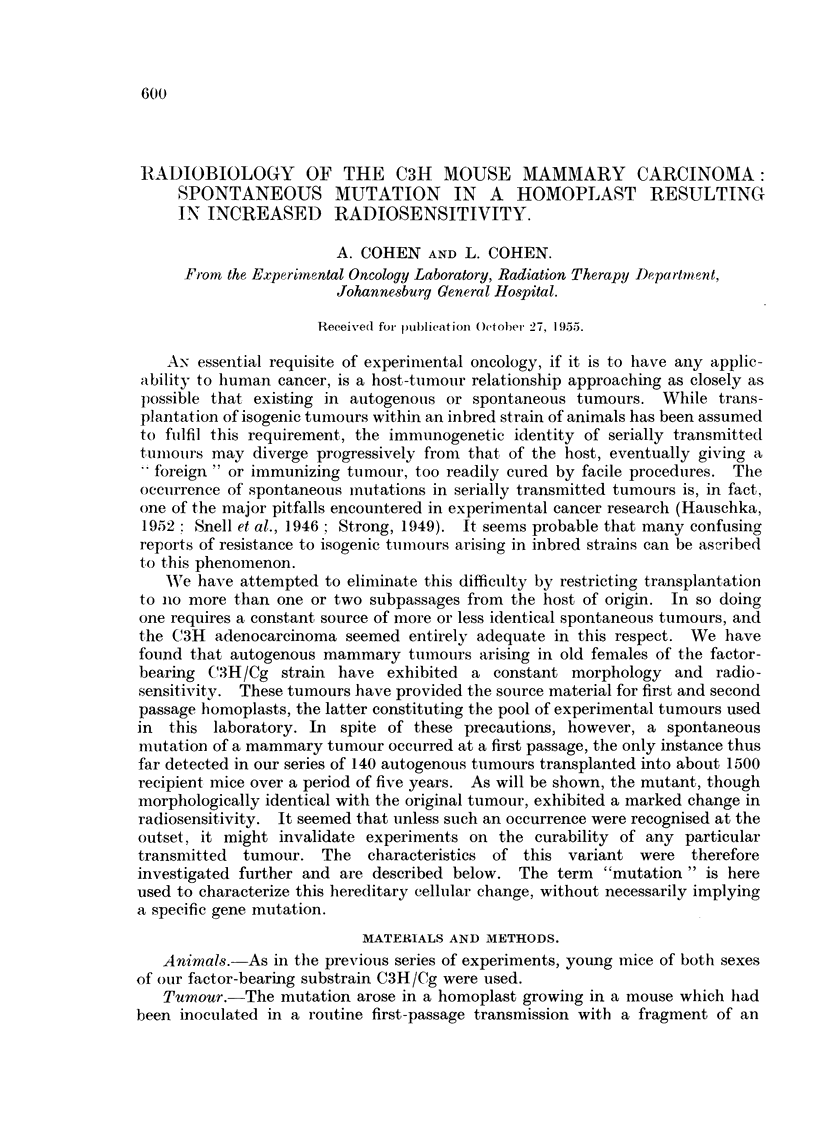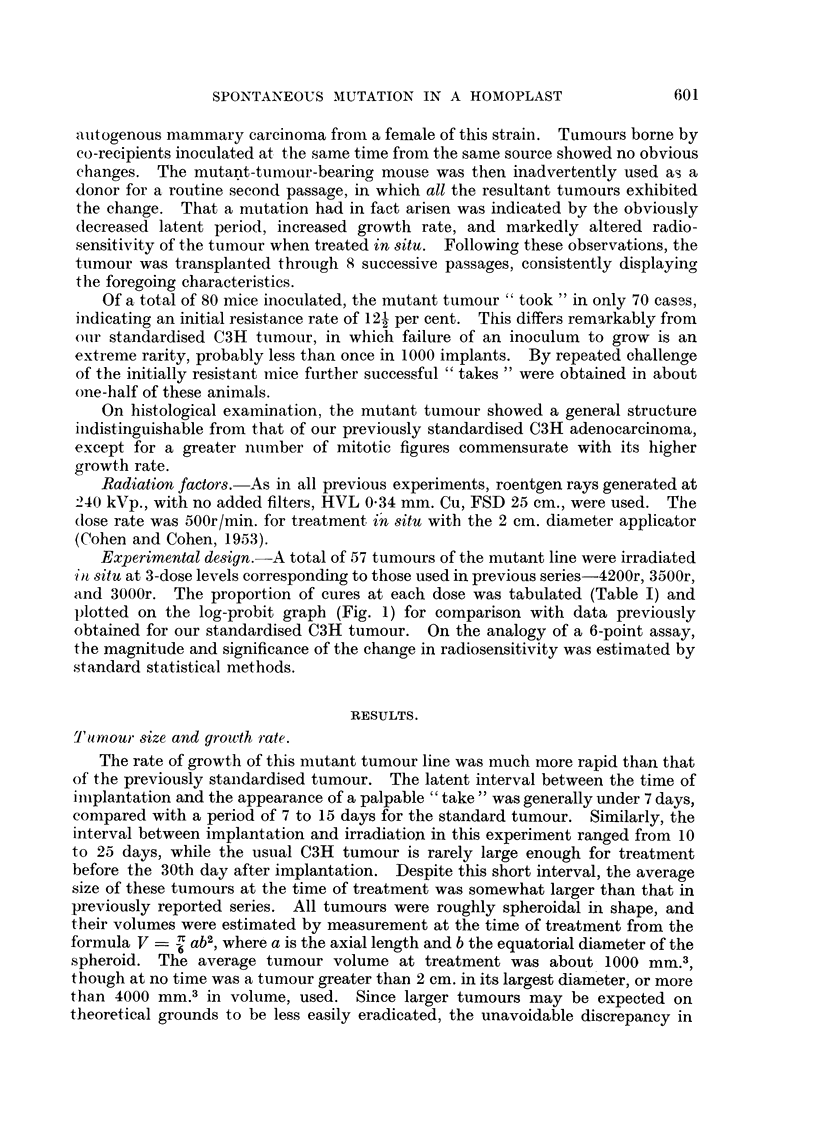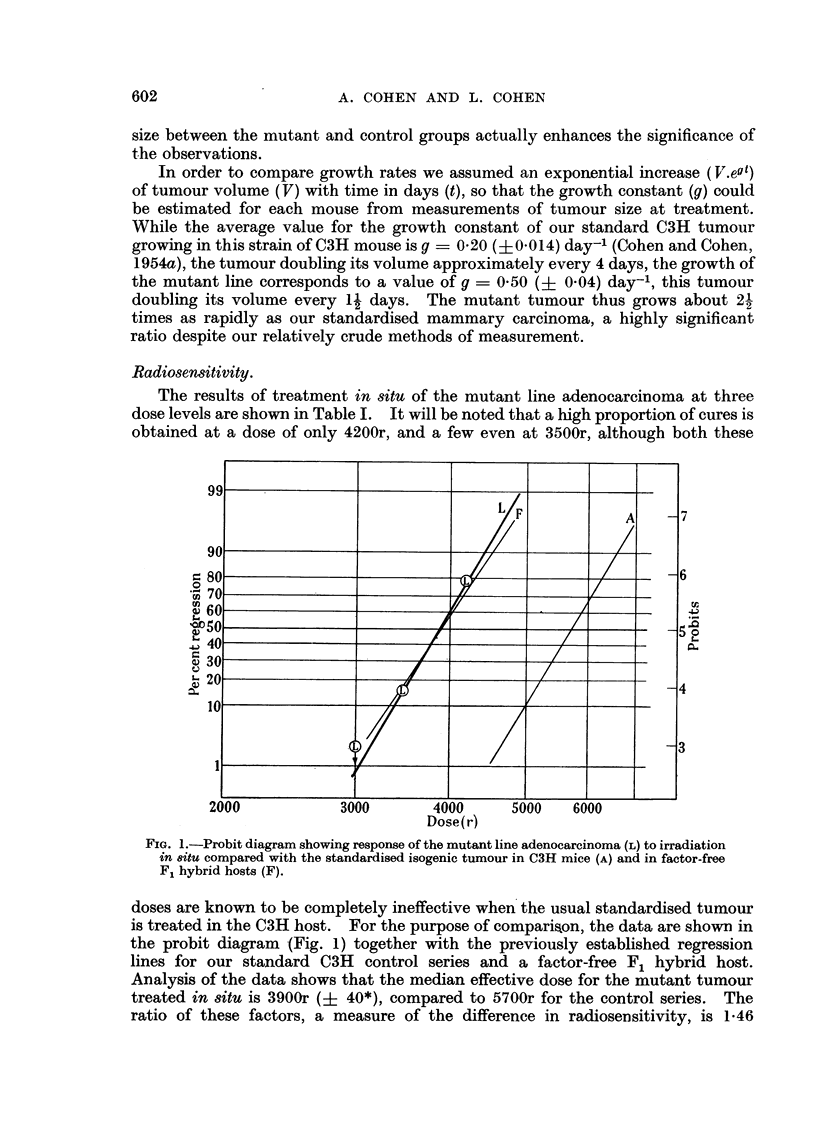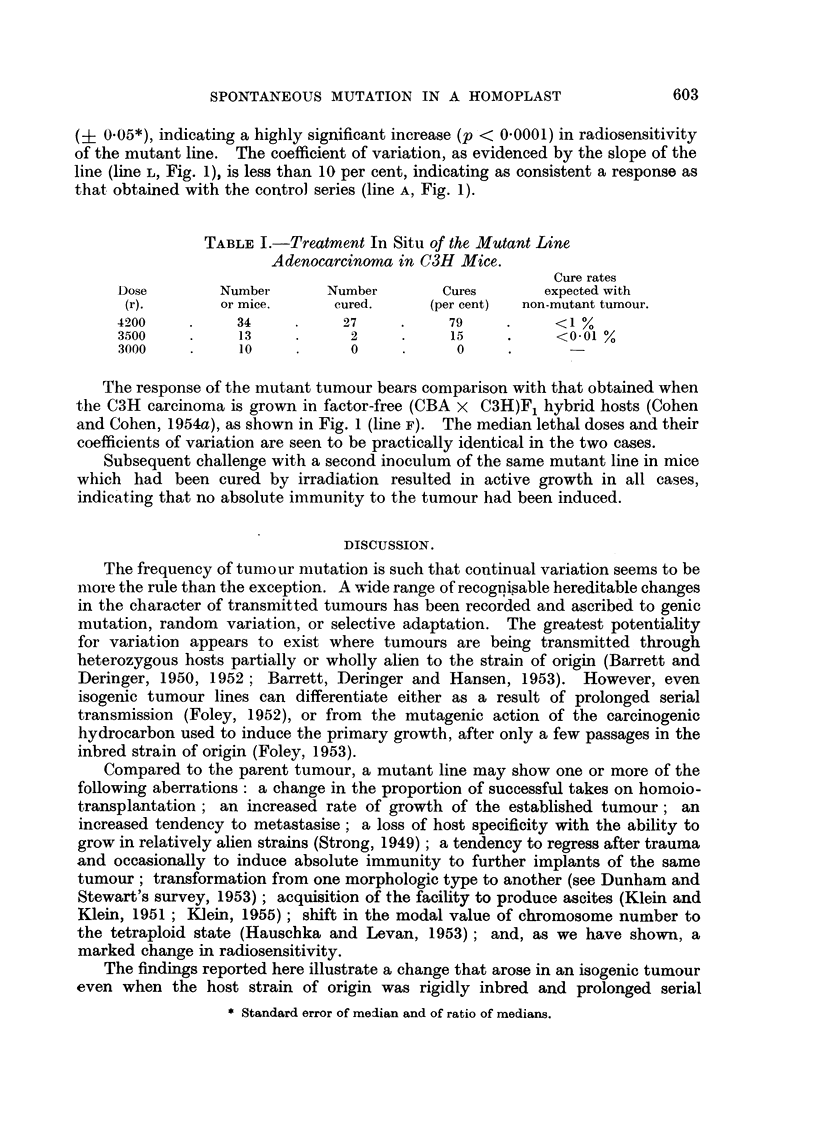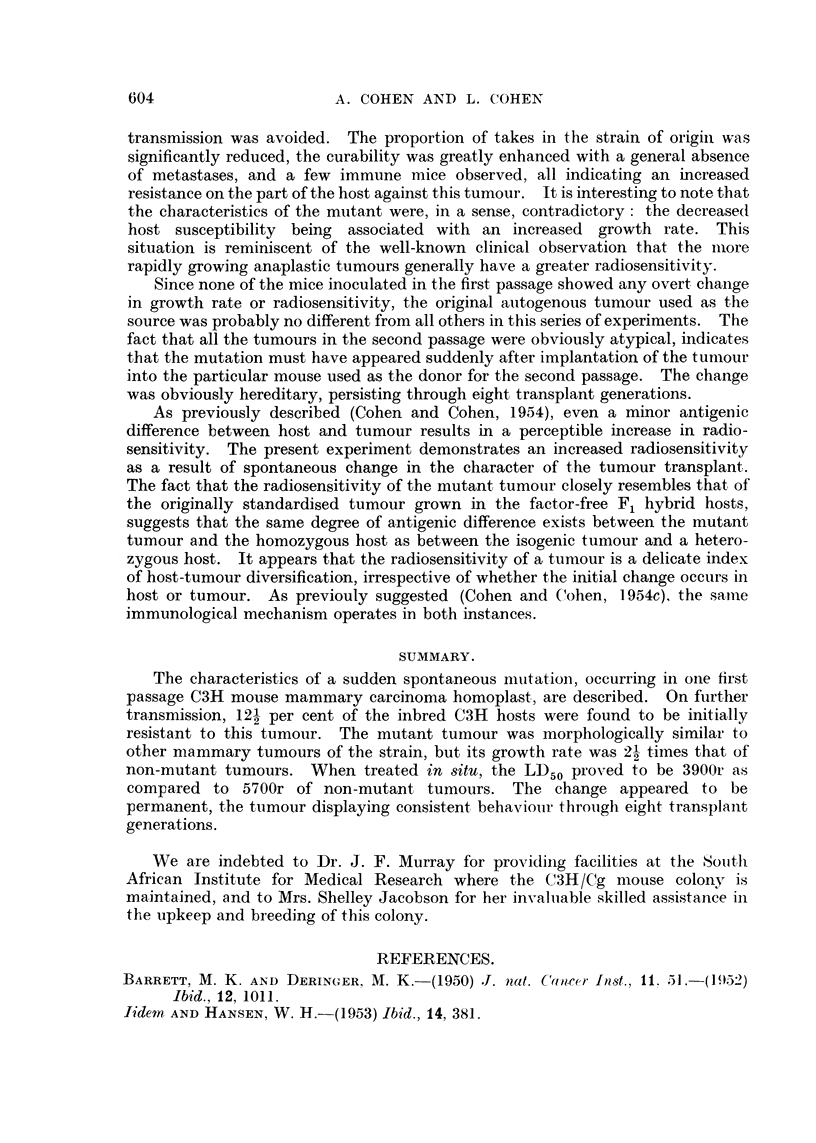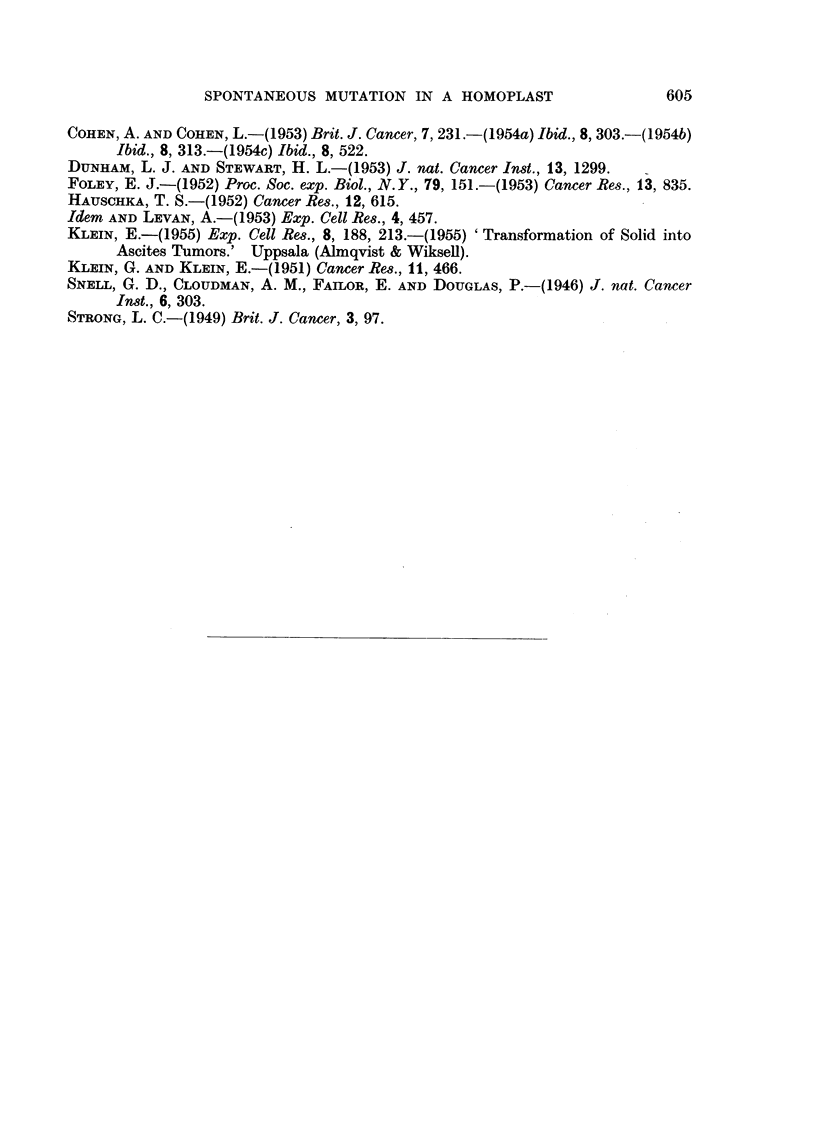# Radiobiology of the C3H Mouse Mammary Carcinoma: Spontaneous Mutation in a Homoplast Resulting in Increased Radiosensitivity

**DOI:** 10.1038/bjc.1955.61

**Published:** 1955-12

**Authors:** A. Cohen, L. Cohen


### RADIOBIOLOGY OF THE C3H MOUSE MAMMARY CARCINOMA: SPONTANEOUS MUTATION IN A HOMOPLAST RESULTING IN INCREASED RADIOSENSITIVITY.

A. COHEN AND L. COHEN.

*From the Experimental Oncology Laboratory, Radiation Therapy Department, Johannesburg General Hospital.*

Received for publication October 27, 1955.

An essential requisite of experimental oncology, if it is to have any applicability to human cancer, is a host-tumour relationship approaching as closely as possible that existing in autogenous or spontaneous tumours. While transplantation of isogenic tumours within an inbred strain of animals has been assumed to fulfil this requirement, the immunogenetic identity of serially transmitted tumours may diverge progressively from that of the host, eventually giving a "foreign" or immunizing tumour, too readily cured by facile procedures. The occurrence of spontaneous mutations in serially transmitted tumours is, in fact, one of the major pitfalls encountered in experimental cancer research (Hauschka, 1952; Snell *et al.*, 1946; Strong, 1949). It seems probable that many confusing reports of resistance to isogenic tumours arising in inbred strains can be ascribed to this phenomenon.

We have attempted to eliminate this difficulty by restricting transplantation to no more than one or two subpassages from the host of origin. In so doing one requires a constant source of more or less identical spontaneous tumours, and the C3H adenocarcinoma seemed entirely adequate in this respect. We have found that autogenous mammary tumours arising in old females of the factor-bearing C3H/Cg strain have exhibited a constant morphology and radiosensitivity. These tumours have provided the source material for first and second passage homoplasts, the latter constituting the pool of experimental tumours used in this laboratory. In spite of these precautions, however, a spontaneous mutation of a mammary tumour occurred at a first passage, the only instance thus far detected in our series of 140 autogenous tumours transplanted into about 1500 recipient mice over a period of five years. As will be shown, the mutant, though morphologically identical with the original tumour, exhibited a marked change in radiosensitivity. It seemed that unless such an occurrence were recognised at the outset, it might invalidate experiments on the curability of any particular transmitted tumour. The characteristics of this variant were therefore investigated further and are described below. The term "mutation" is here used to characterize this hereditary cellular change, without necessarily implying a specific gene mutation.

### MATERIALS AND METHODS.

*Animals.*—As in the previous series of experiments, young mice of both sexes of our factor-bearing substrain C3H/Cg were used.

*Tumour.*—The mutation arose in a homoplast growing in a mouse which had been inoculated in a routine first-passage transmission with a fragment of an autogenous mammary carcinoma from a female of this strain. Tumours borne by co-recipients inoculated at the same time from the same source showed no obvious changes. The mutant-tumour-bearing mouse was then inadvertently used as a donor for a routine second passage, in which *all* the resultant tumours exhibited the change. That a mutation had in fact arisen was indicated by the obviously decreased latent period, increased growth rate, and markedly altered radiosensitivity of the tumour when treated *in situ*. Following these observations, the tumour was transplanted through 8 successive passages, consistently displaying the foregoing characteristics.

Of a total of 80 mice inoculated, the mutant tumour " took " in only 70 cases, indicating an initial resistance rate of $12\frac{1}{2}$ per cent. This differs remarkably from our standardised C3H tumour, in which failure of an inoculum to grow is an extreme rarity, probably less than once in 1000 implants. By repeated challenge of the initially resistant mice further successful " takes " were obtained in about one-half of these animals.

On histological examination, the mutant tumour showed a general structure indistinguishable from that of our previously standardised C3H adenocarcinoma, except for a greater number of mitotic figures commensurate with its higher growth rate.

*Radiation factors.*—As in all previous experiments, roentgen rays generated at 240 kVp., with no added filters, HVL 0·34 mm. Cu, FSD 25 cm., were used. The dose rate was 500r/min. for treatment *in situ* with the 2 cm. diameter applicator (Cohen and Cohen, 1953).

*Experimental design.*—A total of 57 tumours of the mutant line were irradiated *in situ* at 3-dose levels corresponding to those used in previous series—4200r, 3500r, and 3000r. The proportion of cures at each dose was tabulated (Table I) and plotted on the log-probit graph (Fig. 1) for comparison with data previously obtained for our standardised C3H tumour. On the analogy of a 6-point assay, the magnitude and significance of the change in radiosensitivity was estimated by standard statistical methods.

### RESULTS.

### *Tumour size and growth rate.*

The rate of growth of this mutant tumour line was much more rapid than that of the previously standardised tumour. The latent interval between the time of implantation and the appearance of a palpable "take" was generally under 7 days, compared with a period of 7 to 15 days for the standard tumour. Similarly, the interval between implantation and irradiation in this experiment ranged from 10 to 25 days, while the usual C3H tumour is rarely large enough for treatment before the 30th day after implantation. Despite this short interval, the average size of these tumours at the time of treatment was somewhat larger than that in previously reported series. All tumours were roughly spheroidal in shape, and their volumes were estimated by measurement at the time of treatment from the formula $V = \frac{\pi}{6} ab^2$, where $a$ is the axial length and $b$ the equatorial diameter of the spheroid. The average tumour volume at treatment was about 1000 mm.$^3$, though at no time was a tumour greater than 2 cm. in its largest diameter, or more than 4000 mm.$^3$ in volume, used. Since larger tumours may be expected on theoretical grounds to be less easily eradicated, the unavoidable discrepancy in size between the mutant and control groups actually enhances the significance of the observations.

In order to compare growth rates we assumed an exponential increase ($V.e^{gt}$) of tumour volume ($V$) with time in days ($t$), so that the growth constant ($g$) could be estimated for each mouse from measurements of tumour size at treatment. While the average value for the growth constant of our standard C3H tumour growing in this strain of C3H mouse is $g = 0{\cdot}20\ (\pm 0{\cdot}014)\ \text{day}^{-1}$ (Cohen and Cohen, 1954*a*), the tumour doubling its volume approximately every 4 days, the growth of the mutant line corresponds to a value of $g = 0{\cdot}50\ (\pm\ 0{\cdot}04)\ \text{day}^{-1}$, this tumour doubling its volume every $1\frac{1}{2}$ days. The mutant tumour thus grows about $2\frac{1}{2}$ times as rapidly as our standardised mammary carcinoma, a highly significant ratio despite our relatively crude methods of measurement.

*Radiosensitivity.*

The results of treatment *in situ* of the mutant line adenocarcinoma at three dose levels are shown in Table I. It will be noted that a high proportion of cures is obtained at a dose of only 4200r, and a few even at 3500r, although both these

FIG. 1.—Probit diagram showing response of the mutant line adenocarcinoma (L) to irradiation *in situ* compared with the standardised isogenic tumour in C3H mice (A) and in factor-free $F_1$ hybrid hosts (F).

doses are known to be completely ineffective when the usual standardised tumour is treated in the C3H host. For the purpose of comparison, the data are shown in the probit diagram (Fig. 1) together with the previously established regression lines for our standard C3H control series and a factor-free $F_1$ hybrid host. Analysis of the data shows that the median effective dose for the mutant tumour treated *in situ* is 3900r ($\pm$ 40*), compared to 5700r for the control series. The ratio of these factors, a measure of the difference in radiosensitivity, is 1·46

($\pm$ 0·05*), indicating a highly significant increase ($p < 0{\cdot}0001$) in radiosensitivity of the mutant line. The coefficient of variation, as evidenced by the slope of the line (line L, Fig. 1), is less than 10 per cent, indicating as consistent a response as that obtained with the control series (line A, Fig. 1).

TABLE I.—*Treatment* In Situ *of the Mutant Line Adenocarcinoma in C3H Mice.*

| Dose (r). | Number or mice. | Number cured. | Cures (per cent) | Cure rates expected with non-mutant tumour. |
|---|---|---|---|---|
| 4200 | 34 | 27 | 79 | <1 % |
| 3500 | 13 | 2 | 15 | <0·01 % |
| 3000 | 10 | 0 | 0 | — |

The response of the mutant tumour bears comparison with that obtained when the C3H carcinoma is grown in factor-free (CBA $\times$ C3H)$F_1$ hybrid hosts (Cohen and Cohen, 1954*a*), as shown in Fig. 1 (line F). The median lethal doses and their coefficients of variation are seen to be practically identical in the two cases.

Subsequent challenge with a second inoculum of the same mutant line in mice which had been cured by irradiation resulted in active growth in all cases, indicating that no absolute immunity to the tumour had been induced.

### DISCUSSION.

The frequency of tumour mutation is such that continual variation seems to be more the rule than the exception. A wide range of recognisable hereditable changes in the character of transmitted tumours has been recorded and ascribed to genic mutation, random variation, or selective adaptation. The greatest potentiality for variation appears to exist where tumours are being transmitted through heterozygous hosts partially or wholly alien to the strain of origin (Barrett and Deringer, 1950, 1952 ; Barrett, Deringer and Hansen, 1953). However, even isogenic tumour lines can differentiate either as a result of prolonged serial transmission (Foley, 1952), or from the mutagenic action of the carcinogenic hydrocarbon used to induce the primary growth, after only a few passages in the inbred strain of origin (Foley, 1953).

Compared to the parent tumour, a mutant line may show one or more of the following aberrations : a change in the proportion of successful takes on homoio-transplantation ; an increased rate of growth of the established tumour ; an increased tendency to metastasise ; a loss of host specificity with the ability to grow in relatively alien strains (Strong, 1949) ; a tendency to regress after trauma and occasionally to induce absolute immunity to further implants of the same tumour ; transformation from one morphologic type to another (see Dunham and Stewart's survey, 1953) ; acquisition of the facility to produce ascites (Klein and Klein, 1951 ; Klein, 1955) ; shift in the modal value of chromosome number to the tetraploid state (Hauschka and Levan, 1953) ; and, as we have shown, a marked change in radiosensitivity.

The findings reported here illustrate a change that arose in an isogenic tumour even when the host strain of origin was rigidly inbred and prolonged serial

* Standard error of median and of ratio of medians.

transmission was avoided. The proportion of takes in the strain of origin was significantly reduced, the curability was greatly enhanced with a general absence of metastases, and a few immune mice observed, all indicating an increased resistance on the part of the host against this tumour. It is interesting to note that the characteristics of the mutant were, in a sense, contradictory : the decreased host susceptibility being associated with an increased growth rate. This situation is reminiscent of the well-known clinical observation that the more rapidly growing anaplastic tumours generally have a greater radiosensitivity.

Since none of the mice inoculated in the first passage showed any overt change in growth rate or radiosensitivity, the original autogenous tumour used as the source was probably no different from all others in this series of experiments. The fact that all the tumours in the second passage were obviously atypical, indicates that the mutation must have appeared suddenly after implantation of the tumour into the particular mouse used as the donor for the second passage. The change was obviously hereditary, persisting through eight transplant generations.

As previously described (Cohen and Cohen, 1954), even a minor antigenic difference between host and tumour results in a perceptible increase in radiosensitivity. The present experiment demonstrates an increased radiosensitivity as a result of spontaneous change in the character of the tumour transplant. The fact that the radiosensitivity of the mutant tumour closely resembles that of the originally standardised tumour grown in the factor-free $F_1$ hybrid hosts, suggests that the same degree of antigenic difference exists between the mutant tumour and the homozygous host as between the isogenic tumour and a heterozygous host. It appears that the radiosensitivity of a tumour is a delicate index of host-tumour diversification, irrespective of whether the initial change occurs in host or tumour. As previouly suggested (Cohen and Cohen, 1954*c*), the same immunological mechanism operates in both instances.

### SUMMARY.

The characteristics of a sudden spontaneous mutation, occurring in one first passage C3H mouse mammary carcinoma homoplast, are described. On further transmission, 12½ per cent of the inbred C3H hosts were found to be initially resistant to this tumour. The mutant tumour was morphologically similar to other mammary tumours of the strain, but its growth rate was 2½ times that of non-mutant tumours. When treated *in situ*, the $LD_{50}$ proved to be 3900r as compared to 5700r of non-mutant tumours. The change appeared to be permanent, the tumour displaying consistent behaviour through eight transplant generations.

We are indebted to Dr. J. F. Murray for providing facilities at the South African Institute for Medical Research where the C3H/Cg mouse colony is maintained, and to Mrs. Shelley Jacobson for her invaluable skilled assistance in the upkeep and breeding of this colony.

### REFERENCES.

BARRETT, M. K. AND DERINGER, M. K.—(1950) *J. nat. Cancer Inst.*, **11**, 51.—(1952) *Ibid.*, **12**, 1011.

*Iidem* AND HANSEN, W. H.—(1953) *Ibid.*, **14**, 381.

COHEN, A. AND COHEN, L.—(1953) *Brit. J. Cancer*, **7**, 231.—(1954*a*) *Ibid.*, **8**, 303.—(1954*b*) *Ibid.*, **8**, 313.—(1954*c*) *Ibid.*, **8**, 522.
DUNHAM, L. J. AND STEWART, H. L.—(1953) *J. nat. Cancer Inst.*, **13**, 1299.
FOLEY, E. J.—(1952) *Proc. Soc. exp. Biol., N.Y.*, **79**, 151.—(1953) *Cancer Res.*, **13**, 835.
HAUSCHKA, T. S.—(1952) *Cancer Res.*, **12**, 615.
*Idem* AND LEVAN, A.—(1953) *Exp. Cell Res.*, **4**, 457.
KLEIN, E.—(1955) *Exp. Cell Res.*, **8**, 188, 213.—(1955) 'Transformation of Solid into Ascites Tumors.' Uppsala (Almqvist & Wiksell).
KLEIN, G. AND KLEIN, E.—(1951) *Cancer Res.*, **11**, 466.
SNELL, G. D., CLOUDMAN, A. M., FAILOR, E. AND DOUGLAS, P.—(1946) *J. nat. Cancer Inst.*, **6**, 303.
STRONG, L. C.—(1949) *Brit. J. Cancer*, **3**, 97.